# Oral Bacteria: Friends and Foes?

**DOI:** 10.3390/pathogens12111319

**Published:** 2023-11-06

**Authors:** Jinzhi He, Lei Cheng, Alessandra Nara de Souza Rastelli, Dongmei Deng

**Affiliations:** 1 State Key Laboratory of Oral Diseases & National Center for Stomatology & National Clinical Research Center for Oral Diseases, West China Hospital of Stomatology, Sichuan University, Chengdu 610041, China; 2 Department of Cariology and Endodontics, West China Hospital of Stomatology, Sichuan University, Chengdu 610041, China; 3 Department of Restorative Dentistry, School of Dentistry, Sao Paulo State University—UNESP, Araraquara 14801-903, Brazil; 4 Department of Preventive Dentistry, Academic Centre for Dentistry Amsterdam, University of Amsterdam and Vrije Universiteit Amsterdam, 1081 LA Amsterdam, The Netherlands

The oral cavity is an ideal niche for microbial prosperity due to its stable temperature, suitable pH, and continuous nutrient supply. The oral microbiome is the second largest and most diverse microbiome after the gut microbiome, containing more than 700 species including bacteria, fungi, viruses, and protozoa [1,2]. The oral microbiota colonizes both the hard dental surfaces and the soft mucosal tissues, forming exceptionally complex communities named oral biofilms. This community could be pathogenic (foes of hosts) or beneficial (friends of the host). Some of them are even opportunistic, switching from friends to foes and vice versa in response to the environmental fluctuation. “War and peace” occurs every single moment among microbial residents within the oral biofilm, as well as between the microbial collective and the host [3]. Only when symbiosis is reached among them can oral and systemic health be achieved [4]. In this Special Issue “Oral Bacteria: Friends and Foes”, we have compiled 11 papers including not only systematic/scoping reviews, but also original research articles, aiming to gather state-of-the-art knowledge on oral biofilms and oral ecology for further translational research.

During and after formation, biofilms experience both endogenous and exogenous stresses, which may change the composition and pathogenicity of the microbial community [5,6]. The interactions among microbes are among crucial endogenous factors modulating biofilms. In this Special Issue, taking eukaryotic *Candida albicans* and prokaryotic *Staphylococcus aureus* as examples, Hu et al. (contribution 1) showed that active inter-kingdom bacteria–fungi interactions within biofilms augmented the severity of cutaneous abscess and peritonitis in murine models. The potential mechanism might be that *C. albicans* promoted the growth of *S. aureus* and enhanced the expression of bacterial virulence. Moreover, the co-existence of *C. albicans* and *S. aureus* provided survival privilege for both types of cells against the antibiotic’s challenges. Interestingly, Li et al. [7] provided a good explanation of how an exogenous factor, nutrient supply, may influence biofilms. This study found that a limited supply of vitamin B3 (nicotinic acid, niacin, NA) inhibited the growth of *Candida glabrata* (a predominant fungus in biofilms related to denture stomatitis) but increased its adhesion to denture acrylic surface [7]. It is well known that microbial reproduction and adhesion are necessary for biofilm formation [8]. This study showed not only how exogenous stress influenced cellular behaviors, but also how the regulatory system of *C. glabrata* accordingly responded to environmental fluctuations to ensure its predominance in biofilms.

Hosts benefit from microbial symbiosis, but can be jeopardized by dysbiosis. It is the microbial ecology, rather than individual pathogens alone, that determines the onset and progression of a disease [9,10]. In order to elucidate the etiology of these kinds of poly-microbial infections, a comprehensive understanding of the differences in the microbiome composition between patients and healthy controls is critical [11]. In this Special Issue, changes in the oral microbiome are investigated in patients with systematic diseases such as childhood obesity, type 2 diabetes mellitus (T2D), and hidradenitis suppurativa (HS). Childhood obesity is a global pandemic causing increased morbidity and premature death. Leme et al. (contribution 2) showed that *Lactobacillus acidophilus* and *Lactobacillus gasseri* were more prevalent in the saliva of eutrophic kids with early childhood caries, while these two species were mainly detected in caries-free toddlers in the group with obesity. These findings suggested that nutritional status could modulate the behavior of lactobacilli. It seems that *L. gasseri* had a dual function; it was an indicator of caries in the eutrophic group but a commensal in the obesity group. T2D patients are known to be at a higher risk of dental caries [12]. Cena et al. (contribution 3) retrieved, screened, and meta-analyzed studies on the salivary microbiota of adults with T2D compared to adults without T2D. They found that the relative abundances of acidogenic and aciduric bacteria were higher in T2D patients than the non-T2D glycemic controls. In addition, the authors showed a trend of depletion of Proteobacteria and enrichment of Firmicutes (such as *Lactobacillus* and *Veillonela*) in T2D patients, although a better quality of evidence was required. HS is a type of chronic inflammatory dermatosis. Jastrząb et al. (contribution 4) showed that HS changed both the “quantity” (i.e., the total bacterial load) and the “quality” (i.e., the microbial composition) of the subgingival microbiome as compared to their healthy controls. In addition, the subgingival microbiome of HS patients was similar to that of periodontitis patients, which partly explained why HS patients are vulnerable to periodontitis. 

Despite ample evidence on the strong associations between a dysbiotic oral microbiome and systematic diseases, the question on how oral microbes influence the progress of systematic diseases has not been well answered. The hypothesis is that oral microbes could lead to distal inflammation through either activating a remote inflammatory response or ectopic colonization in distal tissues [13]. Shen and coauthors made an effort to unravel this question (contribution 5). Through the transplantation of periodontal pathogens to germ-free mice, they demonstrated that the oral colonization of periodontal pathogens activated immune responses in distal organs such as the spleen and colon in a species- and tissue-dependent manner. These data provided a crucial clue to decipher the mechanisms underlying the oral microbiome and systematic diseases.

Oral microbiome dysbiosis represents a serious threat not only to human beings but also to animals. In this Special Issue, we include a veterinary study that aimed to determine the differences between the microbial distribution of healthy dogs and that of dogs with periodontitis. The data showed that *Treponema denticola* was only detected in dogs with periodontitis, suggesting its essential role in canine periodontal disease (contribution 6).

Biofilm-related diseases such as dental caries and periodontitis are global burdens. Biofilm recalcitrance is a consequence of complex physical and biological biofilm properties with various microbial genetic and molecular factors [14]. Consequently, biofilm infection cannot be easily controlled solely through treatment with antibiotics or "single magic bullet" approaches [14]. The development of novel anti-biofilm strategies is urgent and challenging. This Special Issue includes several studies exploring these novel strategies. Although the chemical structure may be unresolved, natural products show biological activities against biofilms, making them promising therapies for biofilm infection. Saha et al. (contribution 7) showed that extracts of industrial sweet orange waste could reduce the viable bacteria count in both *Streptococcus mutans* planktonic culture and single-species biofilm. Furthermore, these extracts could synergize with chlorhexidine during anti-septicity. Similarly, another natural product, *Cymbopogon citratus* essential oil, was shown to enhance the anti-biofilm effect of chlorhexidine (contribution 8). Photodynamic therapy (PDT) produces reactive oxygen species (ROS) from light-sensitive compounds or structures (also named photosensitizers) in the presence of oxygen, making it responsible for killing microbes and breaking down biofilm matrices [15]. Two key elements of PDT, the photosensitizer and oxygen, are investigated in this Special Issue. de Oliveira et al. (contribution 9) proposed a novel photosensitizer, an extract from Brazilian green propolis. They found that photoactivation in the presence of 1% Brazilian green propolis extract significantly reduced the viability of *S. mutans* and *C. albicans* in both single- and dual-species biofilms without discoloration of the dental material or cellular cytotoxicity. The critical role of the other element in PDT, oxygen, has recently been demonstrated. Nie et al. [16] showed that the absence of oxygen could greatly impair the anti-biofilm efficacy of PDT. The additional H_2_O_2_ could enhance the efficacy of PDT, but in a photosensitizer-dependent manner [16]. These findings are of great clinical significance, especially when using PDT in periodontitis treatment, because a periodontal pocket creates an anaerobic environment for oral microbes and biofilms. In addition, Besegato et al. (contribution 10) found that PDT could be used for cavity decontamination, which effectively inactivated the residual bacteria in dentin without causing microstructural or chemical damages. Hence, PDT can be a powerful tool for the minimally invasive treatment of dental caries. Intelligent materials (also named smart materials) react to various external stimuli or environmental changes by rearranging their molecular structure and adapting their functionality accordingly [17]. The pH drop due to the metabolic activity of oral biofilms is a direct cause of dental caries. As a novel intelligent pH-responsive material, Dodecylmethylaminoethyl methacrylate (DMAEM) can be protonated under acidic conditions and can then play an antimicrobial role. Shi et al. (contribution 11) showed the pH-dependent anti-caries effect of DMAEM-modified resin adhesive. Since biofilms accumulating on the interface of dental material and tooth hard tissues (where the adhesive is located) is a major reason for secondary caries, this finding suggested that DMAEM-modified resin adhesive is a promising material in reducing the occurrence of secondary caries.

Altogether, the information provided in the articles of this Special Issue outline the regulation and pathogenesis mechanisms of oral biofilms, the potential roles of oral biofilms in determining host health, and the development of novel anti-biofilm therapies. These data contribute to our understanding to better control and maintain a healthy oral ecology.
